# EVALUATING PROGRAMS WITH OLDER ADULTS AND CAREGIVERS AS PARTNERS IN PATIENT EDUCATION

**DOI:** 10.1093/geroni/igad104.1578

**Published:** 2023-12-21

**Authors:** Sara Murphy

**Affiliations:** University of North Texas Health Science Center, Fort Worth, Texas, United States

## Abstract

The Geriatrics Workforce Enhancement Program at the University of North Texas Health Science Center has several programs where patients, older adults, caregivers and healthcare stakeholders are incorporated directly into the educational experience or provide feedback to inform the design of educational programs. In addition to the program design, there are evaluation considerations related to incorporating these important stakeholders to best capture the impact of this group. The goal of evaluation research has become more and more important in program design, especially with interdisciplinary programs and programs involving multiple stakeholder groups. Evaluation research is the assessment of outcomes and information that will inform future decision making. These stakeholders are important partners and have an important impact on program design and research outcomes, which is something that should be measured along the way. The University of North Texas Health Science Center Community Advisory Council, Infection Control Advocate and Resident Education (ICARE), Dementia Education, and Seniors Assisting in Geriatric Education are programs that will be included in this evaluation discussion. For these projects, we included evaluation components to keep the patient’s voice at the center of education planning, delivery, and improvement. This presentation will discuss the evaluation of impact in these programs, discuss barriers to measurement, and report early results. One key finding is the lack of systematic measurement tools available and the need for additional tools that can be used across settings where patients are part of the education program design.

